# An association between cancer type and delirium incidence in Japanese elderly patients: A retrospective longitudinal study

**DOI:** 10.1002/cam4.5069

**Published:** 2022-07-26

**Authors:** Kentaro Yamato, Ai Ikeda, Motoki Endo, Ronald Filomeno, Kosuke Kiyohara, Ken Inada, Katsuji Nishimura, Takeshi Tanigawa

**Affiliations:** ^1^ Department of Public Health, Graduate School of Medicine Juntendo University Tokyo Japan; ^2^ Faculty of International Liberal Arts Juntendo University Tokyo Japan; ^3^ Department of Food Science Otsuma Women's University Tokyo Japan; ^4^ Department of Psychiatry Tokyo Women's Medical University Tokyo Japan

**Keywords:** cancer, delirium, longitudinal study

## Abstract

**Objective:**

There is not a known elevated prevalence of delirium in older adult cancer patients. However, it is unknown if the incidence of delirium varies by cancer type among older adult patients. Therefore, this study aimed to examine the association between the incidence of delirium and cancer type among older adult patients using a Japanese hospital‐based administrative claims database.

**Methods:**

A total of 76,868 patients over 65 years of age or older, first diagnosed with cancer on an initial date of hospitalization between April 2008 and December 2019, were included in this retrospective longitudinal study. Delirium was defined by the World Health Organization's International Statistical Classification of Diseases and Related Health Problems (ICD‐10) codes or antipsychotic medication use. Cox proportional hazard models were performed to estimate the risk of delirium incidence according to 22 cancer types during the one‐year hospitalization period.

**Results:**

The incidence rates of delirium were 17.1% for men and 15.3% for women. Compared to gastric cancer, the risk of delirium was significantly higher for pancreatic cancer (HR: 1.26, 95% CI: 1.11–1.42 for men; HR: 1.27, 95% CI: 1.11–1.45 for women), leukemia (HR: 1.24, 95% CI: 1.09–1.41 for men; HR: 1.20, 95% CI: 1.03–1.41 for women), and oropharyngeal cancer (HR: 1.30, 95% CI: 1.10–1.54 for men; HR: 1.32; 95% CI: 1.02–1.72 for women) after adjusting for age, initial hospitalization year, antipsychotic medications, and surgery.

**Conclusions:**

As compared to gastric cancer, patients with pancreatic cancer, leukemia, oropharyngeal cancer were found to have a higher risk of developing delirium. Our study findings suggested that the risk of delirium incidence may vary by cancer type.

## Graphical abstract

The incidence rates of delirium were 17.1% for men and 15.3% for women with cancer. Compared to gastric cancer, the risk of delirium was significantly higher for pancreatic cancer, leukemia, and oropharyngeal cancer.

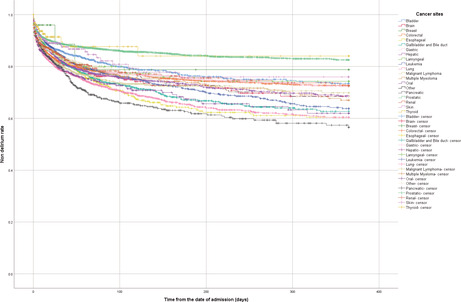

## 1 INTRODUCTION

Delirium is a common symptom in patients with cancer, regardless of the stage. The prevalence ranges from 10% to 31% at in‐patient admission.^1^ One previous study reported that up to 42% of advanced cancer patients were diagnosed with delirium at hospital admission, and 45% developed delirium while admitted.^2^ However, the incidence and prevalence of delirium among cancer patients has not been well established.^3^

Delirium is a neuropsychiatric syndrome characterized by an acute onset of deficits in attention, consciousness, and cognition.^4^ Predisposing factors for developing delirium include advanced age, surgery, cognitive impairment, low albumin level, dehydration, infection, hypoxemia, bone metastases, hematological malignancies, and usage of medications such as steroids, opioids, or benzodiazepines.^4^, ^5^ Although the pathophysiological mechanisms of the development of delirium through these predisposing factors are not clear,^6^ patients with advanced stages of cancer are at an increased risk of developing delirium due to surgery, medications, cancer progression, and comorbidities.^2^

Additionally, delirium may be caused by an elevation of cytokines (i.e., interleukin [IL]‐1, IL‐6, and IL‐8), interferon, and tumor necrosis factor.^7^ Levels of IL‐6 may promote hyperactive delirium^8^ and are significantly elevated in lung, breast,^9^ and colorectal cancers.^10^ Immunohistochemistry assays have also shown that IL‐8 is significantly expressed in gastric cancer tissues.^11^ The onset of delirium depends on the type of cancer due to distinct differences in the expression of inflammatory markers. Furthermore, ApoE2 contributes to cell growth in colorectal cancer patients and is a potential contributor to the development of delirium.^7^, ^12^ Diabetes mellitus is also a known risk factor for both pancreatic cancer and delirium.^7^, ^13^ Thus, the risk of developing delirium may also differ by cancer type via metabolic disorders or levels of ApoE.

Dehydration and prescription drugs use, such as steroids and opioids, are common among patients with cancer.^4^, ^14^ Dehydration is often caused by chemotherapy and is a known risk factor of delirium.^15^ Steroids are included in many chemotherapy regimens,^16^ and opioids are administered to patients with moderate to severe pain.^17^ Additionally, opioids including morphine, hydromorphone, oxycodone, fentanyl, and methadone are often used for pain management and can induce neurotoxicity that may, in turn, lead to delirium.^18^, ^19^

Advanced age is a significant risk factor for developing delirium due to age‐related decline in regional cerebral blood flow, neuronal loss, and neurotransmitter systems.^20^ The prevalence of delirium among people living in the community is low overall (1%–2%), but the risk increases among the older adult community population (8%–17%).^7^ The prevalence of delirium in older adult patients with advanced lung cancer was found to be much higher (43%).^21^ A meta‐analysis has provided evidence that delirium in older adult patients is associated with poor health outcomes such as mortality, institutionalization, and dementia.^22^ Older adult patients with cancer were found to have a higher prevalence of delirium, placing a heavy burden on the patients themselves and their families.^23^

However, to our knowledge, only one study has examined the association of delirium with cancer type and advanced age.^5^ In Japan, the proportion of the population aged 65 years or older was 28.6% by 2021 (https://www.stat.go.jp/english/info/news/20211228.html). Therefore, it is important to examine the risk of delirium symptoms developing in older patients, according to cancer type. If the incidence of delirium can be stratified by cancer type, this study will provide useful information to assist healthcare professionals in preventing the onset of delirium symptoms among the growing number of elderly cancer patients. Based on a prior study showing that the risk of delirium development differs according to sex,^24^ the present analyses were stratified by sex. The data were drawn from the largest nationwide database of administrative claims in Japanese hospitals (Medical Data Vision databases [MDV], https://www.mdv.co.jp).

## 2 METHODS

### 2.1 Data source

Data from patients with cancer under inpatient care were extracted from claim forms of the MDV database in Japan between April 2008 and December 2019. The database contains data from about 24 million in‐ and outpatients (about 19% of the Japanese population) from 360 large hospitals that have adopted the Diagnostic Procedure Combination/Per‐Diem Payment System, which comprise about a fifth of all hospitals in Japan. The MDV contains anonymized patient characteristics, diagnoses, medical expenses, procedures, and drug prescriptions. Patient information from within the same hospital can be traced over time. The MDV data were provided by Medical Data Vision, a commercial data service. As the data were anonymized, informed consent was not required, in accordance with the Ethical Guidelines for Medical and Health Research Involving Human Subjects in Japan.

### 2.2 Inclusion and exclusion criteria

Patients aged 65 years or older who were first diagnosed with cancer on an initial date of hospitalization between April 2008 and December 2019 and were diagnosed with only one type of cancer were included in the present study. Patients diagnosed with a psychiatric disorder or dementia before the initial date of hospitalization or with multiple cancers during the study period were excluded from the study.

### 2.3 Definition of delirium and cancer

Delirium was defined as any condition identified using any of the relevant World Health Organization International Statistical Classification of Diseases and Related Health Problems (ICD‐10) codes: A812, E512, F050, F101, F102, F109, F111, F112, F119, F121, F122, F129, F131, F132, F139, F141, F142, F149, F151, F152, F159, F161, F162, F169, F181, F182, F189, F191, F192, F199, G92, G934, I673, I674, I678, J108, J118, or P916 (Table S1). Alternatively, we identified delirium based on the prescription of antipsychotic medications (risperidone, quetiapine, haloperidol, olanzapine, perospirone, or tiapride) during the hospital stay (Table S2). Some of the antipsychotic medications (quetiapine, haloperidol, olanzapine) were included based on previous studies.^25^, ^26^ The remainder were included based on the Japanese clinical practice guidelines.^4^

According to a previous study, cancer types were defined using ICD‐10 criteria: bladder cancer (C67), brain and other nervous system cancers (C70, C71, C72), breast cancer (C50), colorectal cancer (C18, C19, C20), esophageal cancer (C15), gallbladder and bile duct cancer (C23, C24), gastric cancer (C16), hepatic cancer (C22), laryngeal cancer (C32), leukemia (C91, C92, C93, C94, C95), lung cancer (C33, C34), malignant lymphoma (C81, C82, C83, C84, C85, C96), multiple myeloma (C88, C90), oral and pharyngeal cancer (C00, C01, C02, C03, C04, C05, C06, C07, C08, C09, C10, C11, C12, C13, C14), ovary cancer (C56), pancreatic cancer (C25), prostatic cancer (C61), renal cancer (C65, C66, C68), skin cancer (C43, C44), thyroid cancer (C73), uterine cancer (C53, C54, C55), and other cancers (C17, C21, C26, C30, C31, C37, C38, C40, C41, C45, C46, C47, C48, C49, C51, C52, C57, C58, C60, C62, C63, C69).^27^

### 2.4 Study design

**FIGURE 1:**
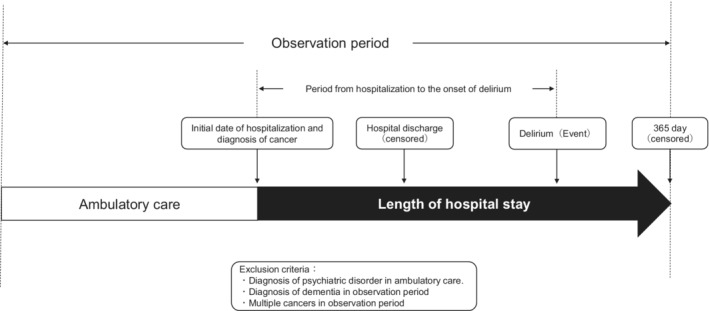
A framework of the present study

This study was designed as a retrospective longitudinal observational cohort study (Figure 1). The data used include those for patients admitted via the emergency ward as well as ambulatory patients. The follow‐up period, in person‐years, was calculated for each type of cancer for a one‐year hospitalization period (365.25 days), from the date of initial hospitalization to the date of delirium onset. The patients were censored at the date of delirium onset, discharge, or after reaching 365 days without delirium, whichever came first. There were no data available for the period after discharge.

### 2.5 Statistical analysis

The patients' demographic characteristics at initial hospitalization were summarized. Kaplan–Meier survival analysis and Cox regression analyses were performed to estimate the hazard ratio (HR) and 95% confidence interval (CI) of delirium incidence during the study period according to cancer type.

All analyses were stratified by sex. The multivariate analysis included age (year), calendar year of initial hospitalization date (year), surgery (yes/no), opioid use (yes/no), benzodiazepine as an antianxiety or hypnotic treatment (yes/no), non‐benzodiazepines (yes/no), and steroid use (yes/no) as potential confounders. Therefore, Model 1 was adjusted for age and calendar year on initial date of hospitalization. Model 2 was further adjusted for the history of surgery. Finally, Model 3 was further adjusted for opioid, benzodiazepine as antianxiety or hypnotics, non‐benzodiazepine and steroid. All analyses were stratified by sex using SAS version 9.4 software (SAS Institute Inc.) and SPSS (IBM). The study protocol was approved by the Juntendo Human Research Ethics Committee (#2020250).

## 3 RESULTS

**FIGURE 2:**
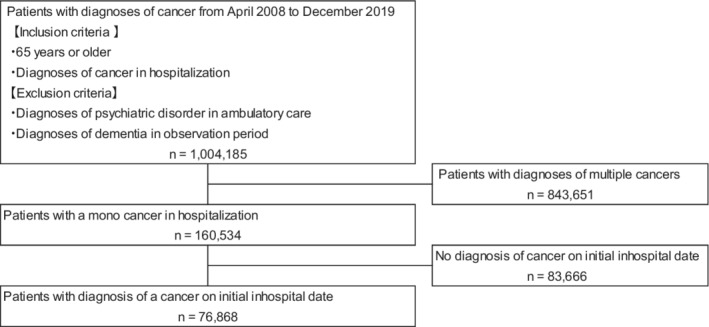
A flow diagram of study patients

Data from a total of 1,004,185 older adult patients with cancer in ambulatory care and without psychiatric disorders or dementia diagnoses were obtained from the MDV database between April 2008 and December 2020. After applying the inclusion criteria, 76,868 patients (44,971 men and 31,897 women) diagnosed with cancer at the initial date of hospitalization were included in the study (Figure 2).

The demographic characteristics of patients were presented in Table 1. The median age of patients was 79 years (interquartile range = 72–84) for men and 81 years (interquartile range = 74–87) for women. The incidence rates of delirium during the follow‐up were 17.1% for men and 15.3% for women. The most common cancers were prostatic cancer (18.2%), gastric cancer (14.6%), and colorectal cancer (14.2%) for male patients; and colorectal cancer (18.5%), gastric cancer (11.6%), and breast cancer (11.3%) for female patients.

**TABLE 1:** The baseline characteristics of patients according to sex

	Total (*N* = 76,868)		Men (*N* = 44,971)		Women (*N* = 31,897)		*p*‐value^a^
	Number	% or median (Inter quartile range)	Number	% or median (Inter quartile range)	Number	% or median (Inter quartile range)	
Age, y			‐	79 (72–84)	‐	81 (74–87)	<0.0001
65–69	10,180	13.2	6600	14.7	3580	11.2	
70–74	12,883	16.7	8345	18.6	4538	14.2	
75–79	14,950	19.4	9341	20.8	5609	17.6	
80–84	16,107	20.9	9534	21.2	6573	20.6	
85–89	13,700	17.8	7387	16.4	6313	19.8	
90–94	6905	8.9	3050	6.8	3855	12.1	
95–99	1895	2.4	649	1.4	1246	3.9	
≧100	248	0.3	65	0.1	183	0.6	
Delirium	12,561	16.3	7686	17.1	4875	15.3	<0.0001
Bladder cancer	2787	3.6	1961	4.4	826	2.6	<0.0001
Brain and other nervous system	939	1.2	492	1.1	447	1.4	0.0001
Breast cancer	3632	4.7	25	0.1	3607	11.3	<0.0001
Colorectal cancer	12,296	16.0	6382	14.2	5914	18.5	<0.0001
Esophageal cancer	1744	2.2	1412	3.1	332	1.0	<0.0001
Gallbladder and Bile duct cancer	3700	4.8	1794	4.0	1906	6.0	<0.0001
Gastric cancer	10,265	13.3	6571	14.6	3694	11.6	<0.0001
Hepatic cancer	5450	7.0	3370	7.5	2080	6.5	<0.0001
Lanryngeal cancer	412	0.5	382	0.8	30	0.1	<0.0001
Leukemia	2906	3.7	1566	3.5	1340	4.2	<0.0001
Lung cancer	7332	9.5	5023	11.2	2309	7.2	<0.0001
Malignant Lymphoma	2831	3.6	1413	3.1	1418	4.4	<0.0001
Multiple Myeloma	1736	2.2	815	1.8	921	2.9	<0.0001
Oral and pharyngeal cancer	1102	1.4	783	1.7	319	1.0	<0.0001
Other	3609	4.7	1986	4.4	1623	5.1	<0.0001
Ovary cancer	382	0.5	‐	‐	382	1.2	<0.0001
Pancreatic cancer	3344	4.3	1491	3.3	1853	5.8	<0.0001
Prostatic cancer	8185	10.6	8181	18.2	‐	‐	<0.0001
Renal cancer	1384	1.8	820	1.8	564	1.8	0.56
Skin cancer	785	1.0	345	0.8	440	1.4	<0.0001
Thyroid cancer	807	1.0	159	0.4	648	2.0	<0.0001
Uterine cancer	1240	1.6	‐	‐	1240	3.9	<0.0001

^a^ Chi‐square test.

The hazard ratios (HRs) and 95% confidence intervals (CIs) for age were 1.03 (95% CI = 1.03–1.04) for men and 1.02 (95% CI = 1.02–1.03) for women, and for surgery these were 1.03 (95% CI = 0.95–1.05) for men and 0.93 (95% CI = 0.87–0.99) for women (not shown in Table 2). Because of its position on the Kaplan–Meier curve, gastric cancer was used as a reference (Figures S1 and S2). Table 2 shows the multivariate HR and CI for each cancer type compared to gastric cancer. After adjusting for age, calendar year of initial hospitalization date, history of surgery, and medications including opioids, benzodiazepines, non‐benzodiazepines, and steroids, the delirium incidences for both men and women were significant as follows: pancreatic cancer (HR = 1.26; 95% CI = 1.11–1.42 for men; HR = 1.27; 95% CI = 1.11–1.45 for women), leukemia (HR = 1.24; 95% CI = 1.09–1.41 for men, HR = 1.20; 95% CI = 1.03–1.41 for women) and oropharyngeal cancer (HR = 1.30; 95% CI = 1.10–1.54 for men, HR = 1.32; 95% CI = 1.02–1.72 for women). Among men, a higher risk of delirium incidence was found in brain and other nervous system cancers (HR = 1.40; 95% CI = 1.14–1.71), lung cancer (HR = 1.31; 95% CI = 1.20–1.43), malignant lymphoma (HR = 1.24; 95% CI = 1.08–1.42), esophageal cancer (HR = 1.21; 95% CI = 1.06–1.39), and hepatocellular carcinoma (HR = 1.15; 95% CI = 1.04–1.27); prostatic cancer had a lower risk of delirium incidence (HR = 0.65; 95% CI = 0.59–0.71). In women, a lower risk of delirium incidence was found for patients with skin cancer (HR = 0.55; 95% CI = 0.39–0.80), breast cancer (HR = 0.69; 95% CI = 0.60–0.79), and bladder cancer (HR = 0.74; 95% CI = 0.59–0.92).

**TABLE 2:** The hazard ratios of delirium according to cancer type and sex

	Men								Women							
	Person‐years	Number of events	Model 1		Model 2		Model 3		Person‐years	Number of events	Model 1		Model 2		Model 3	
			HR	95% CI	HR	95% CI	HR	95% CI			HR	95% CI	HR	95% CI	HR	95% CI
Gastric cancer^a^	1141	1060	1.00	‐	1.00	‐	1.00	‐	606	540	1.00	‐	1.00	‐	1.00	‐
Bladder cancer	341	278	0.87	0.77–1.00	0.87	0.77–1.00	0.90	0.79–1.02	167	91	0.72	0.58–0.90	0.72	0.58–0.90	0.74	0.59–0.92
Brain and other nervous system	113	104	1.27	1.03–1.55	1.27	1.04–1.55	1.40	1.14–1.71	106	62	0.87	0.67–1.13	0.86	0.66–1.12	0.95	0.73–1.24
Breast cancer	5	3	0.66	0.21–2.03	0.65	0.21–2.03	0.68	0.22–2.10	631	323	0.66	0.57–0.75	0.65	0.57–0.75	0.69	0.60–0.79
Colorectal cancer	1169	1074	1.06	0.97–1.15	1.06	0.97–1.15	1.05	0,97–1.15	1085	918	1.05	0.95–1.17	1.06	0.95–1.18	1.05	0.94–1.16
Esophageal cancer	213	259	1.24	1.08–1.42	1.24	1.08–1.42	1.21	1.06–1.39	49	49	1.15	0.86–1.54	1.14	0.85–1.53	1.08	0.81–1.45
Gallbladder and Bile duct cancer	333	356	1.119	0.99–1.26	1.12	0.99–1.26	1.01	0.90–1.14	395	349	1.11	0.97–1.27	1.13	0.98–1.29	1.01	0.89–1.16
Hepatic cancer	640	598	1.16	1.05–1.28	1.16	1.05–1.28	1.15	1.04–1.27	424	303	1.01	0.87–1.16	1.00	0.87–1.15	0.97	0.84–1.12
Laryngeal cancer	66	61	1.03	0.79–1.33	1.03	0.79–1.33	1.08	0.83–1.39	7	1	0.21	0.03–1.51	0.21	0.03–1.50	0.23	0.03–1.61
Leukemia	374	301	1.15	1.01–1.31	1.15	1.01–1.31	1.24	1.09–1.41	291	221	1.12	0.96–1.31	1.14	0.97–1.33	1.20	1.03–1.41
Lung cancer	642	988	1.37	1.25–1.49	1.37	1.26–1.50	1.31	1.20–1.43	328	354	1.14	1.00–1.30	1.12	0.97–1.28	1.08	0.94–1.24
Malignant Lymphoma	230	267	1.19	1.04–1.37	1.20	1.05–1.37	1.24	1.08–1.42	230	217	1.07	0.91–1.25	1.06	0.90–1.24	1.09	0.93–1.28
Multiple Myeloma	189	155	1.11	0.93–1.31	1.11	0.93–1,31	1.09	0.92–1.29	248	162	1.07	0.90–1.28	1.07	0.90–1.28	1.06	0.89–1.26
Oral and pharyngeal cancer	119	159	1.37	1.16–1.62	1.37	1.16–1.62	1.30	1.10–1.54	45	62	1.41	1.09–1.84	1.39	1.07–1.81	1.32	1.02–1.72
Other cancer	248	280	1.07	0.94–1.22	1.07	0.94–1.23	1.06	0.93–1.22	226	217	1.09	0.93–1.27	1.07	0.91–1.25	1.05	0.90–1.23
Ovary cancer	‐	‐	‐	‐	‐	‐	‐	‐	75	64	1.21	0.94–1.57	1.20	0.92–1.55	1.16	0.89–1.51
Pancreatic cancer	224	333	1.46	1.29–1.65	1.46	1.29–1.65	1.26	1.11–1.42	270	388	1.49	1.31–1.70	1.49	1.31–1.70	1.27	1.11–1.45
Prostatic cancer	1646	888	0.62	0.57–0.68	0.62	0.57–0.68	0.65	0.59–0.71	‐	‐	‐	‐	‐	‐	‐	‐
Renal cancer	135	128	1.00	0.84–1.21	1.00	0.84–1.21	1.00	0.84–1.21	95	95	1.17	0.94–1.46	1.17	0.94–1.45	1.15	0.93–1.43
Skin cancer	58	42	0.74	0.54–1.00	0.74	0.54–1.00	0.81	0.60–1.11	61	31	0.51	0.35–0.73	0.51	0.35–0.73	0.55	0.39–0.80
Thyroid cancer	26	14	0.62	0.36–1.04	0.62	0.36–1.04	0.65	0.38–1.09	98	60	0.74	0.57–0.97	0.74	0.57–0.97	0.77	0.59–1.01
Uterine cancer	‐	‐	‐	‐	‐	‐	‐	‐	211	143	0.86	0.71–1.03	0.85	0.71–1.02	0.87	0.72–1.04

*Notes*: Model 1 is adjusted for age, calendar year on initial date of hospitalization. Model 2 is adjusted as in Model 1 + history of sugery. Model 3 is adjusted as in Model 2 + opioid, benzodiazepine as antianxiety or hypnotics, non‐benzodiazepine and steroid.

Abbreviations: CI, confidence interval; HR, hazard ratio.

^a^ Reference category for cancer types.

## 4 DISCUSSION

To our knowledge, this is the first study to report the association between the risk of delirium and type of cancer. In the present study, the risk of developing delirium was higher among those patients with pancreatic cancer, lymphoma, and oropharyngeal cancer than those with gastric cancer, for both men and women. Additionally, when stratified by sex, the risk of developing delirium in men was higher in those with brain and other nervous system cancers, lung cancer, malignant lymphoma, esophageal cancer, and hepatic cancer than in those with gastric cancer. However, similar risks were not found for women.

In the present study, the risk of developing delirium was higher among those patients with cancerous cells close to the central nervous system (CNS), such as brain or oropharyngeal cancer. One of the risk factors for the development of delirium is the presence of intracranial lesions, such as brain tumors.^28^ CNS cancer can cause delirium primary to lesions and secondary to edema or inflammation.^28^ In our study, the risk of delirium in men with CNS cancer (HR = 1.40; 95% CI = 1.14–1.71) was higher than in those with gastric cancer, with no difference found in women (HR = 0.95; 95% CI = 0.73–1.24), probably because meningiomas are more common in women than in men. Meningiomas are the most common type of primary CNS cancer (24%–30%), most are benign^29^ and are considered to have a lower risk of developing delirium.

Overall, all patients with hematological malignancies, such as leukemia, were more likely to develop delirium. Previously, delirium was primarily observed in older adult patients ineligible for intensive chemotherapy regimens.^30^ Because this study focused on older adult patients, it is possible that many subjects ineligible for intensive chemotherapy regimens were included in our study population. Our results also suggest that delirium is more common in patients with hematological malignancies, as found in previous studies.^5^ Patients with acute myeloid leukemia (AML) are susceptible to infections caused by leukopenia, anemia, and thrombocytopenia due to an overgrowth of leukemia cells in the bone marrow, significantly impairing normal hematopoietic function.^31^ In addition, many regimens for hematological malignancies are immunosuppressive and require long‐term treatment, the main side effect of which is infection.^31^ As infections have been shown to have a major impact on the development of delirium,^4^, ^30^ patients treated with an immunosuppressive regimen are more likely to develop delirium due to these side effects. Therefore, surgical treatments for primary gastric cancer are prioritized due to their non‐immunosuppressive methodology, including endoscopic submucosal dissection (ESD), endoscopic mucosal resection (EMR), or gastrectomy.^32^ In the present study, we found that the prevalence of infection among patients with leukemia was 41.1%, higher than 15.9% of the total, with multiple myeloma and malignant lymphoma at 36.8% and 26.8%, and gastric cancer at 12.9% (not shown in table).

Patients with pancreatic cancer had a higher risk of developing delirium compared to those with gastric cancer. Pancreatic cancer is a complex disease; it involves changes in cancer inflammatory components dominated by IL‐6,^33^ which may affect hyperactive delirium.^8^ Additionally, pancreatic cancer is not diagnosed early and many patients may have progressed considerably by the time of diagnosis.^34^ Thus, patients may be at increased risk of delirium due to a strong inflammatory response.

Results show that male patients with lung cancer have a higher risk of developing delirium than patients with gastric cancer. It has been established that there is a gender difference in histological types for lung cancer; men are more likely to develop squamous cell carcinoma (SqCC), whereas women are more likely to have adenocarcinoma (AC).^35^ A Taiwan registry study reported patients with SqCC histology had a lower survival rate than AC patients (univariate: HR = 1.22, CI 95% 1.17–1.28; multivariate: HR = 1.23; CI 95% = 1.17–1.29) and suggested that these lung cancers should be analyzed separately to provide more precise outcomes.^36^ A Japanese cohort study of 12,509 patients with non‐small cell lung cancer after resection also reported the overall survival of women was significantly better than that of men for both AC (5‐year survival rates, 77.7% versus 61.9%, *p* = 0.00) and non‐AC (5‐year survival rates, 59.3% versus 53.1%, *p* = 0.03).^37^ For AC, women have a significantly better prognosis than men for pathologic stage I/II disease. Therefore, men are at higher risk of SqCC, which is more challenging to treat and is often more severe. Additionally, the inflammatory response from tissue invasion may increase the risk of delirium^38^ in men.

The low risk for patients with breast, skin, or prostate cancer to develop delirium may be due to early detection. Prostate‐specific antigen (PSA) is used in routine medical checkups for prostate cancer detection, particularly in Japan.^39^ Additionally, breast cancer screening and the number of early‐stage patients have increased in Japan.^40^ Therefore, we may have found a lower risk of delirium among patients with those types of cancer due to their early detection compared with gastric cancer.

The strength of this study is that it examined the onset of delirium by cancer type using a large hospital database. Diagnosing delirium is complicated, and it is difficult to ensure reliability with the diagnosis of the insurance claims alone, but its reliability could be ensured by citing a study that evaluated the identification of delirium by combining diagnosis (ICD‐10) and medications.^26^ Delirium was common in men and in those with hematological malignancies, as seen in previous studies.^5^ This study is the first to associate the risk of developing delirium by cancer type. No previous studies have been stratified by cancer type. This study showed that delirium increases in pancreatic cancer, lung cancer, brain tumors, and hematologic cancer.

## 5 STUDY LIMITATIONS

This study has several limitations. First, it is retrospective and observational in nature and uses only one medical insurance claims database. The MDV database includes patients treated at large acute‐care hospitals in Japan. Therefore, the findings may not be generalizable to patients outside of large hospitals that have adopted the Diagnostic Procedure Combination/Per‐Diem Payment System. Second, because we used the MDV database, there was a lack of data linkage among medical care facilities. Thus, some of the patients included in this study may have been previously diagnosed with cancer at another facility. Third, the severity of the cancer could not be confirmed; thus, it was impossible to know whether the cancer had progressed since diagnosis. Fourth, because of the insurance reimbursement system in use in Japan (https://www.hospital.or.jp/pdf/14_20110928_01.pdf), physicians often use off‐label prescription of antipsychotics to treat delirium symptoms. We therefore identified the onset of delirium symptoms based on both ICD‐10 codes and the administration of antipsychotics. This may limit the reproducibility of this study, since the antipsychotic medications used to treat delirium may differ by country, and their use to treat delirium may be difficult to distinguish from their use to treat other psychotic disorders. Fifth, we were not able to differentiate the subtype of delirium (i.e., hyperactive, hypoactive, mixed motor, or no motor). Finally, the possibility of patients developing psychiatric disorders or dementia after hospital admission could not be completely eliminated, although patients diagnosed with these disorders before the date of initial hospitalization were excluded from the study.

## 6 CLINICAL IMPLICATIONS

This study examined the risk of the onset of delirium in patients with cancer using a large database of administrative claims in Japanese hospitals. Compared to gastric cancer, men with brain and other nervous system cancers, esophageal cancer, hepatocellular carcinoma, leukemia, malignant lymphoma, lung cancer, oropharyngeal cancer, and pancreatic cancer were more likely to develop delirium. Women with leukemia, oropharyngeal cancer, and pancreatic cancer were at increased risk of developing delirium. Patients with pancreatic cancer, leukemia, oropharyngeal cancer had a higher risk of developing delirium overall. The risk of developing delirium depends on the type of cancer. A previous study report has suggested that the risk of delirium can be reduced among hospitalized elderly when the risk factor specific presentative strategies were introduced.^5^ Our study suggested that it is important to consider sex‐ and cancer‐specific preventions for delirium in elderly patients with cancer.

## AUTHOR CONTRIBUTIONS

All authors contributed to the conception of the study design; KY, AI, ME, KK and TT to the data analysis and interpretation; KY, AI to drafting of the manuscript; and KY, AI, ME, RF, KK, KI, KN and TT to revising the manuscript for important intellectual content. All authors reviewed and approved the final draft of this manuscript and agree to be accountable for all aspects of the work.

## CONFLICT OF INTEREST

KI received personal fees from Eisai, Eli Lilly, Janssen, Meiji Seika Pharma, Mitsubishi Tanabe Pharma, Mochida, MSD, Novartis, Otsuka, Shionogi, Sumitomo Pharma, Yoshitomiyakuhin and he received research grant support from Mochida and Sumitomo pharma in the last three years. KN received personal fees from Meiji Seika Pharma, Mochida, Takeda, Yoshitomiyakuhin, Pfizer, Eli Lilly, MSD, Shionogi, Janssen, Eisai, Astellas, Otsuka, Daiichi Sankyo, Nipro, Kissei, Tsumura, Novartis, Mitsubishi Tanabe, and Chugai and he has received research/grant support from Mochida, Takeda, Otsuka, Novartis, Mitsubishi Tanabe, Dainippon Sumitomo, MSD, Eisai, Tsumura, Eli Lilly, GlaxoSmithKline, and Mebix in the last three years. The other authors declare no conflict of interest.

## ETHICS STATEMENT

This study used anonymized data. Because the study used anonymized information, informed consent was not required, in accordance with Ethical Guidelines for Medical and Health Research Involving Human Subjects in Japan. The study protocol was approved by the Juntendo Human Research Ethics Committee (#2020250).

## Supporting information


Figure S1

Figure S2

Table S1

Table S2

## Data Availability

The data set used for analysis in this study is available from MDV, but were used under license for the current study. Restrictions thus apply, and the data are not publicly available. For inquiries about access to the data set used in this study, please contact MDV (https://www.mdv.co.jp).
