# Supplementary figures and images for: An association between cancer type and delirium incidence in Japanese elderly patients: A retrospective longitudinal study
Source: Cancer Med. 2022 Jul 26;12(3):2407–16. doi: 10.1002/cam4.5069 (PMC9939101; doi:10.1002/cam4.5069)

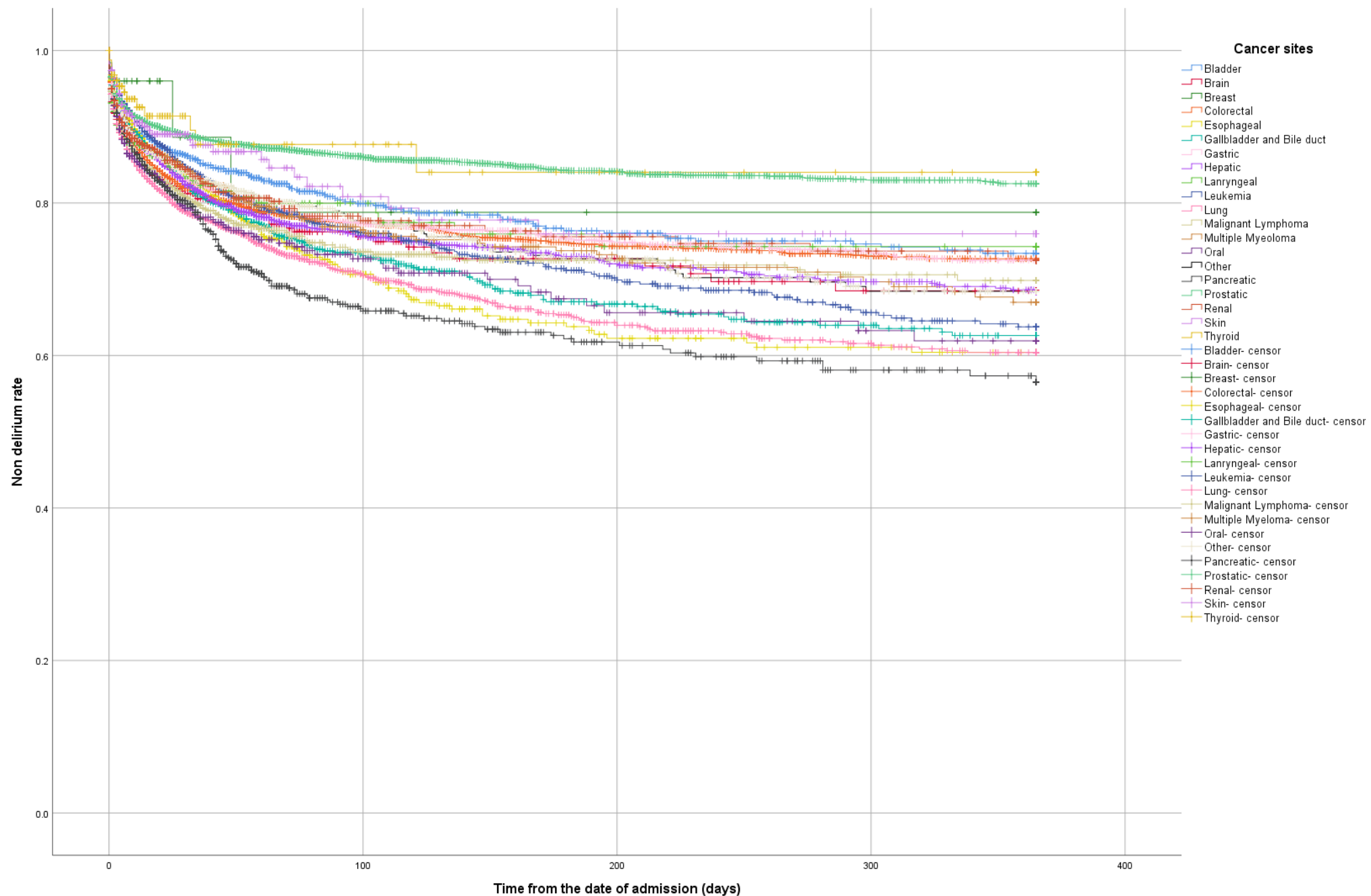

Supplement: Supplementary file 1 — Figure S1 [file CAM4-12-2407-s001.pdf]

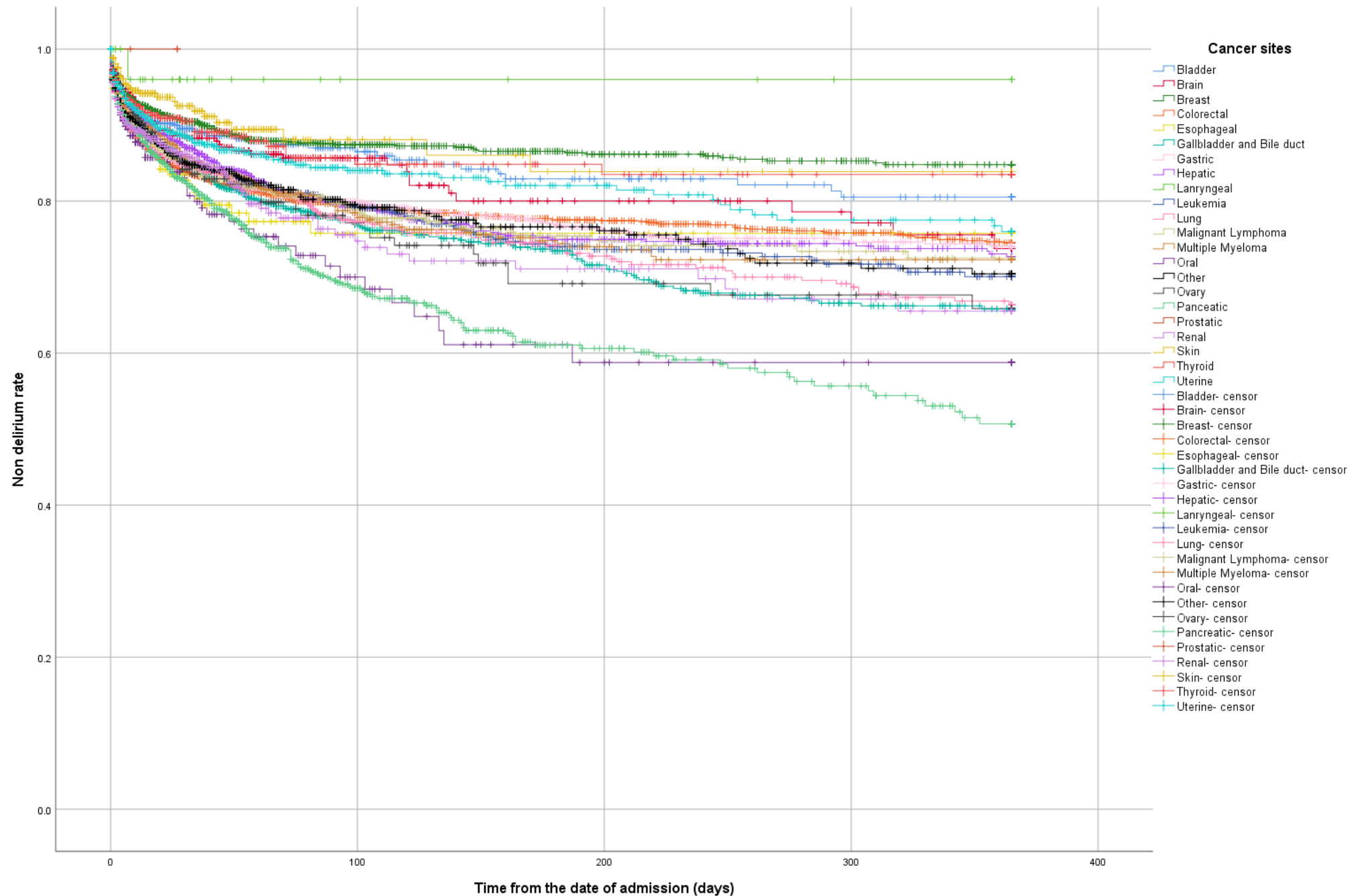

Supplement: Supplementary file 2 — Figure S2 [file CAM4-12-2407-s003.pdf]
