# Supplementary material for: An association between cancer type and delirium incidence in Japanese elderly patients: A retrospective longitudinal study
Source: Cancer Med. 2022 Jul 26;12(3):2407–16. doi: 10.1002/cam4.5069 (PMC9939101; doi:10.1002/cam4.5069)
Supplement: Supplementary file 3 — Table S1 [file CAM4-12-2407-s002.docx]

**Table S1.** The number of patients in the study diagnosed according to the ICD-10 code list for symptoms of delirium.
