# Supplementary material for: An association between cancer type and delirium incidence in Japanese elderly patients: A retrospective longitudinal study
Source: Cancer Med. 2022 Jul 26;12(3):2407–16. doi: 10.1002/cam4.5069 (PMC9939101; doi:10.1002/cam4.5069)
Supplement: Supplementary file 4 — Table S2 [file CAM4-12-2407-s004.docx]

**Table S2.** The number of patients suffering from delirium according to the ATC codes for antipsychotic medications.
